# Passenger lymphocyte syndrome—Epidemiology, pathogenesis, diagnosis, treatment and future directions: A review

**DOI:** 10.17305/bb.2025.12548

**Published:** 2025-07-30

**Authors:** Yingfang Pan, Aiping Zhao, Xiujiao Jiang, Na Zhou, Jing Wang, Changkui Sun, Fang Zhou

**Affiliations:** 1 Blood Group Reference and Research Laboratory, Shandong Blood Center, Jinan, China; 2 The First Clinical Medical School, Shandong University of Traditional Chinese Medicine, Jinan, China; 3 Department of Hematology, the 960th Hospital of the People’s Liberation Army Joint Logistics Support Force, Jinan, China

**Keywords:** Passenger lymphocyte syndrome, PLS, transplantation, epidemiology, graft survival, red blood cell transfusion, blood group incompatibility, hemolytic anemia

## Abstract

Passenger lymphocyte syndrome (PLS) is a hematological complication that can occur following transplantation, characterized by donor-derived memory B lymphocytes producing antibodies against the recipient’s blood cells. This review examines the pathophysiology, diagnostic approaches, and treatment strategies aimed at enhancing clinical management and standardizing therapeutic protocols for PLS. A literature search was conducted using Web of Science and PubMed to identify relevant publications on PLS, resulting in 79 studies. Studies were selected based on predefined criteria, including a focus on human donor-derived alloimmunity, documented blood group antigen-antibody interactions, transplantation context, clinical data on outcomes or management, and methodological validity. Only studies containing actual patient data and substantive discussions about PLS were included. PLS commonly presents as hemolytic anemia, accompanied by elevated lactate dehydrogenase (LDH) levels, indirect hyperbilirubinemia, and reduced haptoglobin levels. Diagnosis is primarily based on clinical manifestations and laboratory tests, including the direct antiglobulin test (DAT) and antibody screening. Differential diagnosis is crucial for excluding drug-induced hemolytic anemia and thrombotic microangiopathy. Current treatment strategies for PLS focus on halting hemolysis and restoring hematological balance. First-line treatment includes donor-compatible red blood cell transfusions and high-dose corticosteroids, while refractory cases may necessitate rituximab or plasmapheresis. Despite advancements in PLS management, challenges persist, including delayed diagnosis due to self-limiting cases and a lack of standardized treatment protocols. Future research should incorporate genomic and proteomic biomarkers for accurate diagnosis and risk prediction. Developing mechanism-driven therapies targeting donor lymphocytes and establishing global consensus frameworks can enhance monitoring, improve graft survival, and optimize transplant recipient outcomes.

## Introduction

Transplantation is recognized as the most effective therapeutic intervention for organ failure, significantly enhancing long-term survival in patients with end-stage organ disease [1]. However, the limited availability of donor organs continues to pose a significant challenge to the advancement of clinical organ transplantation [2]. Recent medical advancements have facilitated the emergence of ABO-incompatible (ABOi) organ transplantation, which has broadened the donor pool and partially alleviated organ shortages [3]. Importantly, ABOi transplantation has demonstrated comparable outcomes between blood group-mismatched and compatible transplants in hematopoietic stem cells, liver, and kidney transplants [4], thus challenging the traditional belief that ABO compatibility is essential for transplant success. Nonetheless, ABOi transplantation introduces distinct challenges. It is frequently associated with hematological complications, such as passenger lymphocyte syndrome (PLS), immune cytopenia, and transplant-associated thrombotic microangiopathy (TA-TMA) [5, 6]. Among these complications, the incidence of PLS has risen, attracting increased attention from experts in transplantation and blood transfusion. This trend can be attributed to the widespread adoption of transplantation technology, particularly the increase in incompatible transplants involving the previously mentioned blood types. Furthermore, advancements in clinical diagnostic techniques, heightened awareness in monitoring, and a better understanding of donor and recipient factors have contributed to this rising incidence. PLS was first proposed by Beck et al. in 1971 as a mechanism related to donor lymphocytes that underlie hemolysis and was initially documented in humans. This phenomenon was observed in a transplant recipient with type A blood who exhibited elevated titres of anti-A antibodies after receiving a type O lung transplant. The term “PLS” was subsequently introduced by Stevens et al. in 1981 [7]. PLS occurs when donor memory B lymphocytes transferred during transplantation produce antibodies that target recipient red blood cells (RBCs), platelets, or other blood components, resulting in complement-mediated hemolysis, thrombocytopenia, or neutropenia [8].

The incidence of PTLS varies significantly based on the type of transplant. Historically, PTLS has been associated with ABO- or Rhesus (Rh)-incompatible solid organ transplants, such as heart, lung, liver, kidney, and small bowel recent evidence has highlighted its growing relevance in hematopoietic stem cell transplantation (HSCT), particularly in ABO-minor mismatched donors [5, 9]. In minor ABO-mismatched transplants (e.g., donor O to recipient blood group A/B), the reported incidence rates range between 14% for liver transplants (LTs) and 20% for renal ones [10, 11]. Although PLS typically resolves spontaneously within three months, severe cases may require interventions such as transfusion support, corticosteroids, plasmapheresis, or the administration of rituximab. Importantly, the manifestations of PLS extend beyond hemolysis; recent studies have linked PLS to severe thrombocytopenia following transplantation, a condition known as transplant-mediated alloimmune thrombocytopenia (TMAT). This is particularly observed in recipients of organs from donors with immune thrombocytopenic purpura (ITP) [12, 13]. Both TMAT and PLS have been documented in various organ transplants, including liver, kidney, and lung transplants [14].

Additionally, donor-derived anti-human leukocyte antigen (HLA) antibodies in multi-organ transplant recipients indicate novel PLS variants, prompting a re-evaluation of its classification [15]. Recent studies have emphasized the systemic effects of PLS, with documented cases involving small bowel, lung, and pancreatic kidney transplants [16–18]. This expansion of the clinical spectrum of PLS, along with its recognition in patients receiving HSCT, necessitates a comprehensive re-assessment of its mechanisms, organ-specific risk factors, and management strategies. This review synthesizes decades of research to clarify the pathophysiology, heterogeneity across transplant types, and evolving therapeutic approaches, ultimately improving diagnostic accuracy and prognostic evaluation.

## Methods

**Figure 1.**
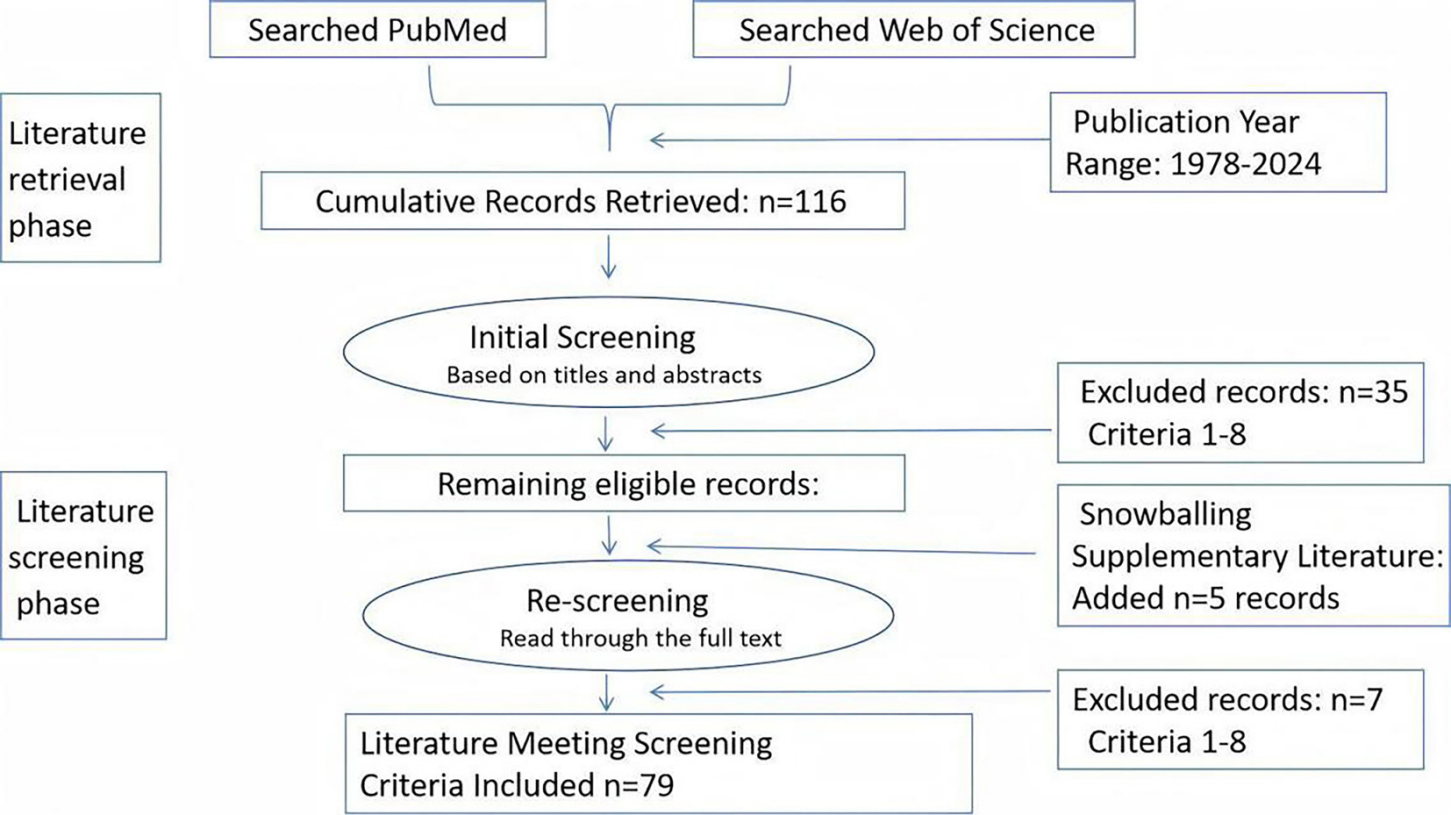
Flowchart of methodological and clinical relevance criteria for inclusion.

To perform a comprehensive literature review, we conducted an extensive search of the Web of Science and PubMed databases using targeted keywords to identify the most relevant publications. The keywords used included “Passenger Lymphocyte Syndrome,” “Blood Group Incompatibility,” and “Transplantation,” covering literature from approximately 1978–2024. The initial search resulted in 116 pertinent articles. A subsequent screening was implemented to refine this selection, concentrating on studies that specifically addressed cases of PLS, rather than solely focusing on other hemolytic conditions related to transplantation. We prioritized research involving human participants with clinical data, thereby excluding animal studies and *in vitro* experiments. This rigorous selection process resulted in the inclusion of 79 articles for our analysis. During the subsequent selection phase, we implemented stringent screening criteria that evaluated the following: 1) a focus on human donor-derived alloimmunity related to PLS; 2) documented blood group antigens and antibodies implicated in the pathogenesis of PLS; 3) type of transplantation (solid organ vs. hematopoietic stem cell); 4) clinical data on outcomes, manifestations, or management strategies; 5) validity of study design (case reports, observational studies, or systematic reviews); 6) inclusion of actual patient data rather than theoretical models; 7) clinical relevance beyond laboratory investigations; and 8) a substantive discussion about PLS rather than peripheral mentions. This comprehensive assessment ensured that the selected studies met rigorous methodological and clinical relevance criteria for inclusion (Figure 1).

### Epidemiological characteristics

The epidemiological characteristics of PLS are intricately associated with the type of transplantation, the degree of blood-type incompatibility, and the immune status of the donor. PLS primarily arises in transplants involving minor ABO blood group incompatibilities. It typically occurs when donors possess blood types O or A/B, while recipients have type A, B, AB, or mismatched Rh blood groups [19]. Among patients undergoing solid organ transplantation, the incidence of PLS varies by organ type, ranging from 9% to 20% in kidney transplants, approximately 40% in LTs, and as high as 70% in heart-lung transplantation patients [20]. Although rare in small intestinal transplantation (ITx), PLS can still lead to significant hemolysis. In patients receiving HSCT, PLS is relatively infrequent but may be initiated by either ABO or non-ABO antibodies, including anti-RhD [21]. Specific age or sex predisposition to PLS remains unestablished. However, children might exhibit increased susceptibility to severe hemolysis due to their relatively immature immune systems, particularly because adults have a fully developed complement system [10]. Additionally, no well-defined regional distribution patterns for PLS have been identified; however, its prevalence may correlate with the frequency of ABO blood group mismatch transplants in different regions. Risk factors for PLS include donors with abundant lymphoid tissue, such as that found in the liver and small intestine, prior sensitization of the donor to the recipient’s RBCs through transfusions or pregnancies, and inadequate immunosuppression, which permits donor lymphocytes to evade immune response control [22, 23].

### Pathogenesis of PLS

**Figure 2.**
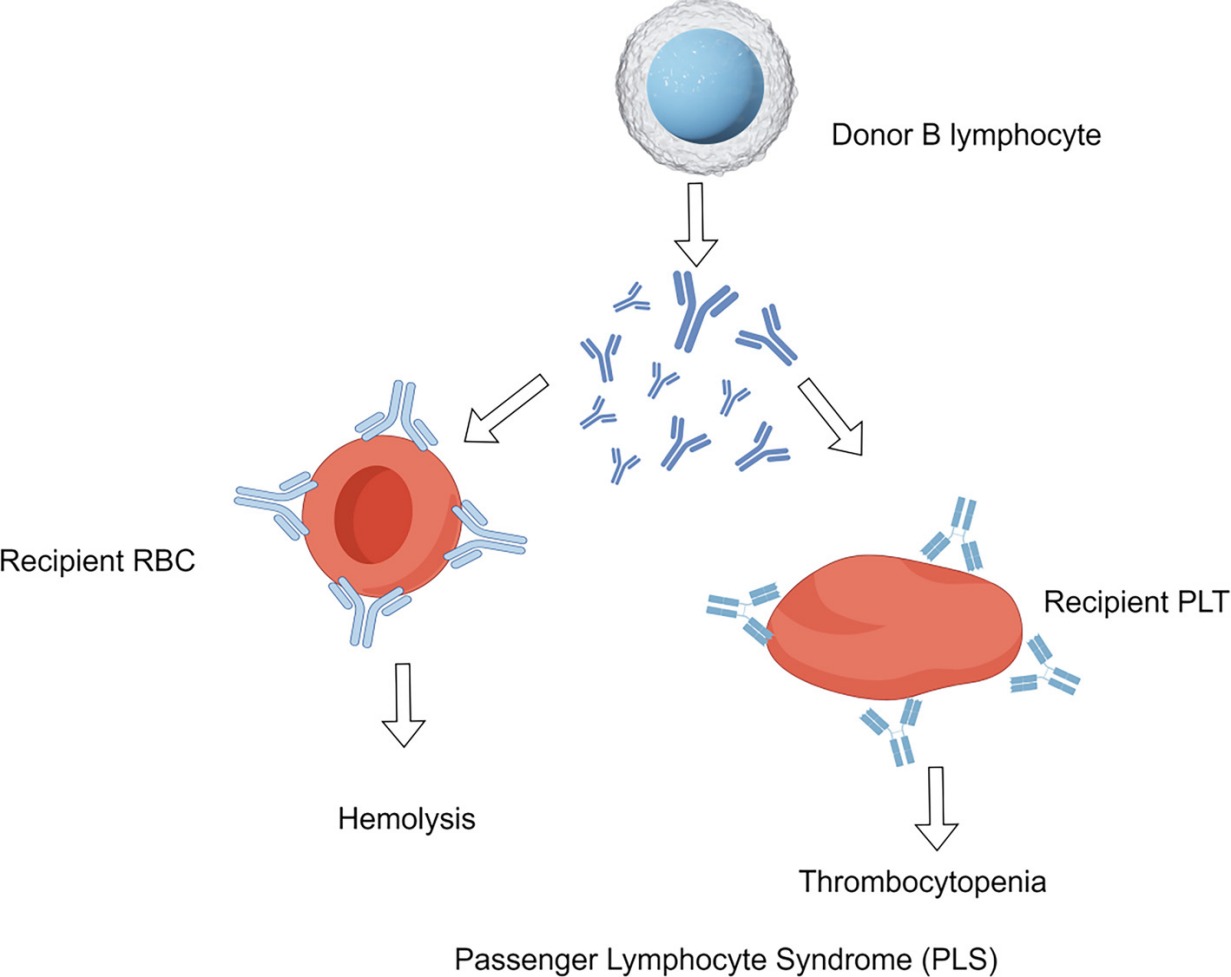
**Pathogenesis of passenger lymphocyte syndrome (PLS).** Passenger B lymphocytes from the graft enter the recipient’s blood and secrete allo-antibodies (anti-A/B, anti-D or platelet-specific). Antibody binding to recipient red blood cells (RBC) and platelets (PLT) triggers complement/Fc-mediated destruction, causing intravascular hemolysis and immune thrombocytopenia; severity rises with higher antibody titres and antigen density. Abbreviations: RBC: Red blood cell; PLT: Platelet.

The pathophysiology of PLS involves the following three key steps (see Figure 2).

Firstly, the transfer of donor lymphocytes occurs as immunocompetent B lymphocytes from the graft migrate into the recipient’s bloodstream. Liver and intestinal grafts, which contain a significant amount of lymphoid tissue, present the highest risk for PLS [8, 24, 25]. Notably, lung transplants demonstrate a high incidence of Rh-associated PLS due to the presence of donor-derived anti-D antibodies [22], while intestinal transplants frequently involve anti-A/B-mediated hemolysis [26].

Second, regarding antibody production: Donor B lymphocytes produce antibodies that target recipient RBC antigens. This includes anti-A/B antibodies in ABOi transplants, anti-D antibodies in cases of Rh incompatibility, and antibodies against platelet antigens. The risk of antibody production is heightened if the donor has previously been alloimmunized with antibodies such as anti-K, anti-Jk(a), and platelet-specific antibodies [27–29]. For example, donors with a history of autoimmune disorders, such as Hashimoto’s thyroiditis, may transfer autoreactive B lymphocytes, potentially resulting in post-transplant acute hemolysis [30].

Third, concerning hemolysis or thrombocytopenia: Antibodies can trigger complement activation (C3d deposition) and/or induce Fc-mediated phagocytosis, resulting in RBC destruction or thrombocytopenia. The severity of hemolysis is correlated with antibody titers and the density of antigens on RBCs [31–33].

### Role of blood group antigens in PLS

PLS is primarily driven by donor-derived antibodies that target recipient RBCs in transplant recipients. It is predominantly associated with antibodies from the ABO blood group, followed by those from the Rh blood group. Additionally, it includes antibodies such as anti-K, Jka, M, and N [34]. ABO minor mismatches (e.g., O→A/B) are the most common triggers; however, non-ABO antibodies (e.g., anti-D, anti-K, anti-E, anti-Jk(a), anti-Kpb, anti-Lea) account for 15%–20% of cases [23, 29]. Notably, PLS occurs in ABOi transplant patients primarily due to minor ABO incompatibility (e.g., O→A). Rarely, bidirectional ABO incompatibility (e.g., A→B) may result in simultaneous donor and recipient antibody-mediated hemolysis [35]. Hemolysis induced by anti-A/B antibodies is typically self-limiting and mild, whereas anti-D-mediated hemolysis generally persists longer, lasting up to 6 months [22, 36]. ABO incompatibility is the primary risk factor, with the highest risk linked to minor ABO mismatches, such as O→A or O→B transplants. Kohl et al. [37] reported an 18.18%–30.77% PLS incidence rate in type A recipients of type O grafts, 5.13% in type B recipients of type O grafts, and 20% in type AB recipients of type O grafts. Rh incompatibility represents a secondary risk factor. Additionally, other blood group systems, such as Kidd and MNS, may contribute to PLS development [38]; however, their relative influence is unclear owing to insufficient data. Furthermore, numerous documented cases of transient lymphatic syndrome associated with Jka antibody-induced hemolysis require significant attention [27, 29]. PLS mediated by non-ABO/Rh antigens has been frequently reported in individual case studies, demonstrating a spectrum of severities that generally correlate with the immunogenic properties of blood group system antigens. Some research has identified instances of anti-HPA-3a/HPA-1a-mediated alloimmune thrombocytopenia in liver, kidney, and combined hepatorenal transplantation, including severe thrombocytopenia induced by liver grafts [14, 39–41]. These findings prompt an investigation into whether antigens on RBCs, platelets, and leukocytes may contribute to PLS. The data indicate that the severity and clinical outcomes of PLS vary across different blood group systems. Notably, ABO incompatibility exhibits the highest incidence when compared to Rh and other blood group systems.

### Diagnosis and differential diagnosis

PLS typically manifests 5–30 days post-transplantation, with the timing of onset influenced by the type of graft and immune interactions. In liver and small bowel transplants, PLS often presents early (days 5–15) due to the high lymphocyte load inherent in these organs. In contrast, ABO-minor mismatched HSCT may exhibit delayed onset (days 20–30) as donor lymphocytes gradually engraft [24, 42–44]. The hallmark clinical features include hemolytic anemia (median hemoglobin [Hb] nadir: 6–8 g/dL) accompanied by biochemical evidence of intravascular hemolysis, such as elevated lactate dehydrogenase (LDH) > 500 U/L, indirect hyperbilirubinemia (> 2 mg/dL), and profoundly reduced haptoglobin (< 0.1g/L) [11, 42, 45, 46]. Organ dysfunction, including hepatic impairment (bilirubin > 5 mg/dL in 40% of LTs) and acute kidney injury (25% of severe cases), further highlights the systemic impact of PLS [47, 48]. Risk stratification identifies key predictors of PLS, including lymphoid-rich grafts (odds ratio [OR]: 4.8, 95% confidence interval [CI]: 2.1–10.9), minor ABO/Rh mismatches (OR: 3.2, 95% CI: 1.8–5.6), and the use of cyclosporine-based regimens (OR: 2.4, 95% CI: 1.3–4.5) [10, 24, 44, 49]. Diagnosis follows a structured algorithm. The initial evaluation centers on unexplained jaundice and a hemoglobin (Hb) drop of greater than 2 g/dL within 24 h, which necessitates serial screenings on days 5, 10, and 15 to monitor Hb trends, LDH, and bilirubin levels [11, 42]. Confirmatory testing begins with the direct antiglobulin test (DAT), which identifies IgG/C3d positivity in 85% of cases. This is complemented by antibody identification to detect donor-derived anti-A/B antibodies, exhibiting a specificity of 98% [36, 50–52]. In cases of uncertainty, advanced techniques such as ABO/Rh genotyping can clarify serological discrepancies (e.g., weak subgroups), while chimerism analysis quantifies donor lymphocytes (with levels exceeding 1% correlating to severity), and HLA antibody screening identifies thrombocytopenic variants [20, 28, 41, 52]. PLS may present atypically, necessitating specialized testing. Non-hemolytic PLS, characterized by positive DAT results without a decline in Hb levels (due to anti-D/M antibodies), requires antibody elution and RBC phenotyping. Sandler et al. proposed a unified classification system that encompasses both hemolytic and non-hemolytic PLS manifestations, aiming to enhance the correlation between clinical presentations and laboratory findings. This system emphasizes the role of lymphocytes in addition to hemolysis [36]. By promoting the identification of subcategories that include both types of manifestations, we offer a more comprehensive representation of the full spectrum of PLS [22, 34]. PLS, defined by a platelet count of less than 50×10^9^/L, is associated with anti-HPA-1a/b antibodies, which can be detected through platelet glycoprotein-specific assays. In cases where concurrent hemolysis and thrombocytopenia are present (similar to Evans syndrome), quantification of CD20+ B cells via peripheral blood flow cytometry or tissue biopsy is warranted.

**Table 1:** Differential diagnosis of passenger lymphocyte syndrome

**Condition**	**Key differentiators**
Drug-induced hemolysis	Temporal relation to drug initiation (e.g., tacrolimus) and drug-dependent antibodies
Thrombotic microangiopathy (TMA)	Schistocytes, reduced ADAMTS13 activity, and microvascular thrombosis
Acute graft-versus-host disease (GVHD)	Skin rash, diarrhoea, negative DAT, and donor chimerism confirmed by biopsy
Pure red cell aplasia (PRCA)	Onset >30 days post-HSCT, reticulocytopenia (<0.1%), normal LDH/haptoglobin, and negative DAT

Abbreviations: DAT: Direct antiglobulin test; HSCT: Hematopoietic stem cell transplantation; LDH: Lactate dehydrogenase.

**Table 2:** Treatment strategies for passenger lymphocyte syndrome (PLS)

**Treatment**	**Timing**	**Efficacy**
Blood transfusion	Severe or refractory hemolysis	Highly effective in most cases
Corticosteroids	If transfusion fails or severe hemolysis	Moderate efficacy, often combined with other therapies
B-Cell and antibody-targeting therapies		
Rituximab (RTX)	After steroid/transfusion failure	∼80% remission (CD20+ B-cell depletion)
IVIG	Acute hemolysis or adjunct to RTX	Short-term reduction in antibody titers
Plasmapheresis (e.g., centrifugal exchange or Glycosorb-ABO immunoadsorption)	Emergent cases (e.g., renal failure)	Rapidly reduces antibody titres (e.g., anti-A IgG from 64→4)
Efgartigimod	Refractory cases	Case reports show synergy with plasmapheresis
Complement-targeting therapies		
Eculizumab	Evidence of complement activation	Anecdotal efficacy (risk of infections)
Sutimlimab	Cold agglutinin-like hemolysis	Theoretical (similar to cold agglutinin disease)
Supportive care	Chronic anemia management	Adjunctive role

“Blood transfusion and supportive care” is considered throughout the entire process of transplantation, while the remaining treatments are all therapeutic measures taken after the transplantation.

**Figure 3.**
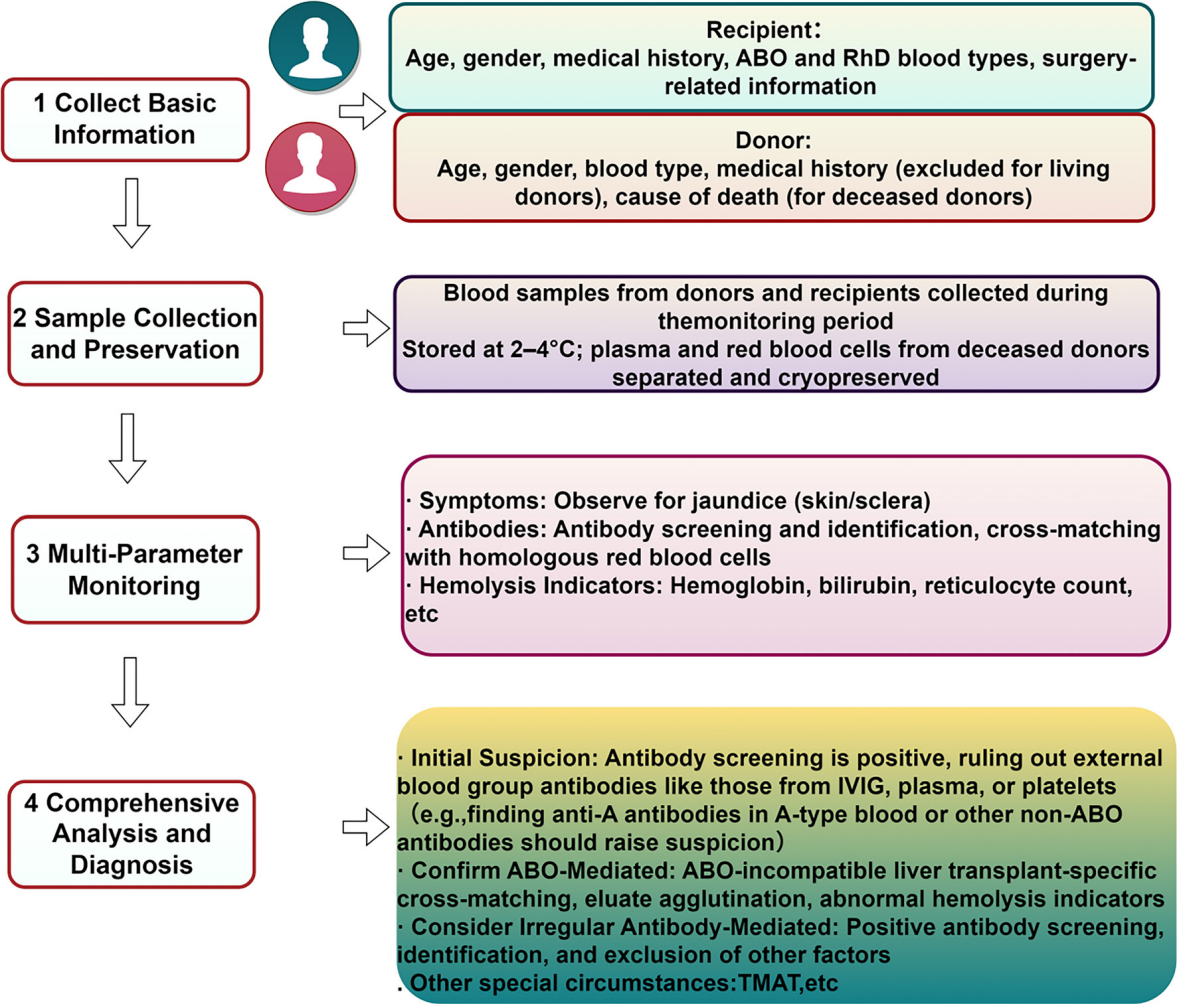
Monitoring process of passenger lymphocyte syndrome (PLS).

A differential diagnosis is essential for excluding conditions mimicking PLS (Table 1). Drug-induced hemolytic anemia, such as that caused by tacrolimus, is characterized by a temporal relationship with drug exposure and the presence of drug-dependent antibodies [53]. Thrombotic microangiopathy (TMA) is a rare but clinically significant syndrome marked by microangiopathic hemolytic anemia, thrombocytopenia, and organ damage due to microcirculatory thrombosis. Key diagnostic indicators that differentiate TMA from other forms of hemolytic anemia include the schistocyte count and evaluation of ADAMTS13 activity. Acute graft-versus-host disease (GVHD) presents with skin rash, diarrhea, and a negative DAT, as confirmed by biopsy and donor chimerism analysis [54, 55]. In HSCT, it is crucial to distinguish between post-transplant complications, PLS, and pure red cell aplasia (PRCA) due to their differing mechanisms and treatments. PRCA, which often follows ABO major mismatch transplants (e.g., A→O), occurs when recipient isoagglutinins inhibit donor erythroid cell differentiation [56]. This leads to reticulocytopenia (<0.1%), normal LDH and haptoglobin levels, absence of erythroid progenitors in the bone marrow, and a negative DAT. Conversely, PLS involves donor antibody-mediated hemolysis, characterized by reticulocytosis, abnormal LDH and haptoglobin levels, and a positive DAT. Treatment for PRCA typically involves reducing immunosuppressive therapy, while PLS requires B-cell-targeted therapies such as rituximab. It is imperative to maintain vigilance during the diagnostic process. Anemia with a positive DAT occurring shortly after HSCT, particularly within 7–21 days, should raise suspicion for PLS. In contrast, anemia that develops beyond 30 days, especially when accompanied by transfusion dependence and reticulocytopenia, strongly suggests PRCA. This diagnosis can be confirmed using flow cytometry, which identifies CD71+ erythroid progenitor cells in the bone marrow. Several diagnostic challenges complicate PLS identification. In approximately 30% of cases, the self-limiting nature of the syndrome may delay confirmation and ABO-identical transplants, particularly when anti-Kell/Jka/Jkb antibodies are overlooked without extended phenotyping [51]. To address these challenges, it is recommended that antibody screening occurs every 48 h for cases of unexplained cytopenia until PLS is confirmed or excluded. This structured approach ensures timely recognition and intervention, especially in high-risk transplantation cohorts. In light of these considerations, we developed a streamlined monitoring protocol from a laboratory perspective (Figure 3). Initially, comprehensive information on both donors and recipients is collected prior to transplantation. For recipients, this data includes age, sex, medical history, ABO and RhD blood types, surgical details (procedure type and timing), and disease-related risk factors. For donors, the information encompasses age, sex, blood type, and medical history (which is not applicable to living donors), with deceased donors requiring additional documentation regarding the cause of death. These foundational data facilitate a risk assessment of disease onset and its influencing factors. During the monitoring period, blood samples from both parties are stored at 2 ^∘^C–4 ^∘^C. In deceased donors, multiple blood samples are collected simultaneously; plasma is separated and stored at −20 ^∘^C, while RBCs are cryopreserved to ensure their availability for follow-up analysis and critical research. Dynamic multi-parameter monitoring involves three key aspects: (1) symptom observation—careful monitoring for jaundice of the skin and sclera, a common indicator of PLS-related hemolysis that reflects disease progression and supports early diagnosis; (2) antibody monitoring—conducting antibody screening and cross-matching with homologous RBCs, with further specificity identification if screening results are positive, effectively distinguishing between ABO antibodies and irregular antibodies; and (3) indicators of hemolysis and anemia—regular assessment of Hb, bilirubin, reticulocyte count, LDH, haptoglobin, and DAT results to evaluate the severity of hemolysis. A decline in Hb levels, elevated bilirubin, or a positive DAT indicates active hemolysis, aiding in the tracking of disease progression. A comprehensive analysis that integrates clinical symptoms, antibody results, and hemolysis trends reveals a correlation between antibody emergence and hemolytic reactions. For example, primary leukocyte sensitization (PLS) is strongly suspected when pre-transfusion testing indicates self-controlled positivity or incompatible cross-matching, excluding alloantibodies. In ABOi transplants, such as from an O donor to an A recipient, ABO antibody-mediated PLS is confirmed if cross-matching with multiple homologous donors fails, eluates agglutinate with homologous RBCs, and hemolysis markers are abnormal. In contrast, irregular antibody-mediated PLS is identified when antibody screening is positive, specificity is confirmed, or other factors are ruled out. Special cases, including unexplained thrombocytopenia, necessitate the evaluation of PLS-associated complications, such as transfusion-related microangiopathic thrombocytopenia (TMAT). This systematic approach facilitates timely diagnosis and tailored management of transplant-related complications.

### Treatment and prognosis

#### Treatment strategies

The current treatment modalities for PLS encompass several key aspects, with management primarily focused on halting hemolysis and restoring hematological stability (Table 2). In blood product management, the emphasis is on transfusing donor-type, compatible, or antigen-negative RBCs—such as type O RBCs for patients with anti-A/B PLS—to avoid exacerbating hemolysis. The transfusion dose is tailored to specific patient conditions, highlighting the importance of early identification and rational product selection. First-line interventions also include high-dose glucocorticoids, such as methylprednisolone at a dosage of 1 mg/kg/day, to suppress donor B cell activity [10, 44, 57, 58]. Furthermore, B-cell targeting and antibody depletion therapy, particularly with rituximab, have shown promise in treating refractory PLS and other autoantibody-associated autoimmune diseases. PLS occurs when donor B-lymphocytes produce antibodies against recipient RBCs, leading to hemolysis [8].

Rituximab, an anti-CD20 antibody, has been demonstrated to be effective in preventing and treating post-transplant lymphoproliferative disorder (PTLD) in various transplant settings [59, 60]. This therapy functions by depleting B-cells, which are precursors to antibody-producing plasma cells [61]. In refractory cases, rituximab achieves remission in 80% of patients by targeting and depleting antibody-producing lymphocytes. In contrast, plasmapheresis rapidly reduces circulating antibodies in patients experiencing life-threatening hemolysis [26, 59, 62, 63]. The effectiveness of treatment for PTLD can be assessed by monitoring changes in ABO antibody titers alongside clinical signs of hemolysis [64].

In a kidney transplant case, the IgG anti-A titre decreased from 64 pre-transplant to 4 post-plasma exchange, coinciding with the resolution of hemolysis. Another report indicated an anti-A1 titre of 8 at diagnosis, with hemolysis resolving following plasma exchange and a transition to group O transfusions. Additionally, one study documented clinical improvement within 28–30 days and antibody clearance occurring within approximately three months, while a case series reported hemolysis resolution ranging from 0–776 days (mean, 148 days) [46]. Elevated transfusion requirements were noted when the titres remained high [37]. Immunosuppressive protocols necessitate individualized formulation. Transplant-related factors, including organ type and blood group mismatch, significantly influence risk and management, with complications typically manifesting 1–3 weeks post-transplantation. Supportive measures, such as erythropoietin, folic acid, and iron supplementation, are essential for mitigating anemia-related complications [11, 65]. Emerging therapies, including eculizumab (an anti-C5 monoclonal antibody) and efgartigimod (a neonatal Fc receptor inhibitor), demonstrate promise in targeting complement activation and enhancing antibody clearance, particularly in cases of PLS complicated by TMA or resistant to conventional therapies [47, 66, 67].

#### Prognostic considerations

PLS is typically self-limiting, resolving within 2–6 weeks as donor lymphocytes are cleared. However, severe cases may result in transfusion dependence (median of 2–8 units), graft dysfunction due to hemoglobinuria-induced injury, or TMA with a 5% incidence [42, 44, 47, 54]. Although mortality remains rare (<5%), it increases with delayed diagnosis, multiorgan failure, or pre-existing comorbidities [12, 68]. Long-term outcomes are generally favorable, with most patients achieving normal graft function after recovery. Early recognition and tailored therapy—optimizing the intensity of immunosuppression to mitigate infection risks—are essential for improving prognosis.

### Clinical classification in PLS

PLS exhibits distinct characteristics in organ transplants, influenced by organ-specific immunological and anatomical factors (Table 3).

**Table 3:** Characteristics of PLS in diverse organ transplantations

**Transplant type**	**Incidence rate**	**Clinical manifestations**	**Treatment and prognosis**	**Special risk factors**
Liver transplantation	Highest incidence, approximately 17.9% in minor ABO incompatibility cases. In pediatric LT, the incidence is 14%, with a higher risk when the recipient is A+ and the donor liver is O+	Marked hyperbilirubinemia, often misdiagnosed. In some cases, elevated indirect bilirubin is the initial manifestation without significant hemoglobin decrease	Generally good prognosis, most cases resolve within 2–3 weeks. Severe hemolysis can lead to graft dysfunction
Kidney transplantation	Approximately 20% in cases of minor ABO incompatibi lity. Overall incidence is lower than that of liver transplantation. Slightly higher risk in living-related kidney donation	Anemia is the primary manifestation. Renal function is usually not affected, but acute kidney injury induced by hemolysis may occur	Usually self-limiting with a low mortality rate, but graft rejection may occur	Donor’s previous immune history, immunosuppressive regimen (influence of cyclosporine), graft abundant in lymphoid tissue
Small intestine transplantation	Low incidence rate but may be potentially severe	More severe hemolysis, often requiring multidiscip linary intervention	Higher mortality rate, possibly related to insufficient immunosuppression intensity	
Lung transplantation	Approximately 18.2%–30.8% in cases of minor ABO incompatibility	May be complicated with thrombotic microangiopathy, significantly increased transfusion requirements	Hemolysis does not affect graft survival, but long-term monitoring of antibody titres is required	
Hematopoietic HSCT	Low incidence rate, accompanied by severe hemolysis, especially in cases of minor ABO incompatibility	Share common features with other transplants such as hemolytic anemia and positive DAT. Symptoms appear within 1–3 weeks after surgery and can be delayed for months	Self-limiting in most cases, low mortality rate	

Abbreviations: DAT: Direct antiglobulin test; HSCT: Hematopoietic stem cell transplantation.

### LTs

The incidence of PLS in LT recipients varies significantly across studies. In ABO minor-incompatible LTs, the incidence ranges from 5% to 20%, with higher rates observed in pediatric patients [69]. A retrospective study of 333 pediatric LT recipients reported a PLS prevalence of 14% (7 out of 51 ABO-compatible cases), particularly among blood group A+ recipients receiving O+ grafts. Similarly, among 1217 adult LT recipients, 12 cases of PLS were identified (10 out of 56 ABO minor-incompatible and 2 out of 147 Rh-incompatible cases) [10]. LTs exhibit the highest incidence of PLS, likely due to the abundance of passenger lymphocytes and the presence of donor-derived B-cell activating factor (BAFF), which enhances B-cell survival and antibody production [70]. Severe hemolysis, often characterized by a median Hb nadir of 6–8 g/dL, frequently necessitates transfusion, while hyperbilirubinemia (> 5 mg/dL) is a common complication.

The overall incidence of PLS in LT recipients demonstrates considerable variability, ranging from 0.5% in large cohorts (14 out of 2772 patients) to as high as 30%–40% or even 100% in smaller case series. The onset of PLS typically occurs 7–14 days post-transplantation; however, cases have been reported to arise immediately after transplantation and up to 120 days later [38].

ABO minor incompatibility is the most frequently reported risk factor in transfusion-related complications, with Rh and other minor antigen mismatches also being observed. Hemolytic anemia is the predominant clinical manifestation, characterized by a decline in Hb levels ranging from 1.5 g/dL to 5.4 g/dL [71], often requiring blood transfusions. Jaundice is commonly reported in affected individuals. Additionally, isolated complications such as thrombocytopenia, renal injury, cardiovascular instability, and graft-related issues [57] have been documented.

### Lung transplants

In lung transplant recipients, PLS has an incidence rate of 0.5% to 2%, presenting as a treatable form of hemolytic anemia with varying degrees of severity. Despite occasional severe complications, most patients experience favorable outcomes. However, reported incidence rates of PLS vary significantly across studies [69]. In lung-specific cohorts, incidence rates range from 0.5% to 2%, while heart-lung transplants demonstrate incidences as high as 70% [38]. In cases of minor ABO mismatches—particularly when an O donor is paired with an A, B, or AB recipient—the incidence rates are 18% to 31% for A, 5% for B, and 20% for AB. Hematological manifestations are the primary clinical feature, with PLS frequently presenting as hemolytic anemia. The severity of this condition varies, with Hb levels dropping modestly (e.g., from 8.3 to 7.4 g/dL) to life-threatening reactions that necessitate blood transfusions, plasmapheresis, or targeted antibody therapy [37].

While rare cases have led to severe complications, including cardiac death, studies generally indicate stable graft function and favorable patient outcomes with appropriate supportive care [38]. Additional factors such as HLA mismatch, the volume of transferred lymphoid tissue, and variations in transplant techniques have been proposed as potential contributors; however, their specific roles remain ambiguous.

### Kidney transplants

A systematic review of 91 cases estimated the incidence of post-transfusion lymphocytopenia syndrome (PLS) to be approximately 20% in ABO-mismatched transplants [65]. This condition typically results in self-limiting anemia, which can be managed with transfusions or erythropoietin; however, graft dysfunction or death occurs infrequently. A case report [10] documented PLS presenting as elevated indirect bilirubin levels without a decline in Hb [45], which resolved after the administration of donor-type RBC transfusion, with no adverse effects on kidney function. In contrast, Dirim et al. [47] reported a refractory PLS case that mimicked cold agglutinin disease. This case did not respond to steroids, intravenous immunoglobulin (IVIG), or rituximab, necessitating immunoadsorption (IA), ultimately resulting in graft loss due to rejection. Another atypical pediatric case of PLS involved gastrointestinal complications, manifesting as ischemic colitis and disseminated intravascular coagulation (DIC), which required colectomy and therapeutic plasma exchange (TPE) [48]. These cases underscore the variability in PLS manifestations, ranging from mild hemolysis to life-threatening organ damage, thereby emphasizing the necessity for timely diagnosis (through direct antiglobulin testing and antibody screening) and individualized treatment approaches (including transfusion, TPE, and IA). Although the majority of PLS cases are self-limiting, refractory instances present significant challenges in treatment, highlighting the need for standardized treatment protocols and further research into prevention and management strategies.

### Small bowel transplants

While the occurrence of PLS in transplantation is well-documented, its incidence following intestinal transplantation (ITx) remains ambiguous. The presence of lymphoid tissue in intestinal grafts heightens the risk of PLS, especially in cases involving ABO minor incompatibility or HLA leukocyte antigen mismatches.

In the field of epidemiology, the incidence of PTLS among ABO minor-incompatible transplant recipients is estimated to be 9% [54]. Key risk factors for PTLS include ABO minor incompatibility, particularly in cases involving O→A/B grafts, which result in donor lymphocyte-mediated production of anti-A/B antibodies [44, 72]. Lymphoid-rich grafts, such as those from small bowel or multivisceral transplants (including the spleen), exhibit a higher risk of PTLS due to the abundance of donor lymphocytes and HLA mismatch. Increased donor-recipient HLA disparity may further exacerbate immune activation [63, 73].

In terms of clinical manifestation and diagnosis, post-transfusion leukocyte antigen sensitization (PLS) typically occurs 7–14 days following transplantation. It is characterized by severe hemolytic anemia, indicated by a drop in Hb levels exceeding 20 g/L, as well as jaundice, elevated LDH, and low haptoglobin levels [44]. A positive DAT can reveal the presence of IgG- or complement-coated rRBCs [74]. In cases of ABO incompatibility, donor-derived antibodies, such as anti-A or anti-B, are commonly observed, whereas non-ABO antibodies, such as anti-M, are rare but have been documented [34]. Furthermore, instances of non-hemolytic PLS, where antibodies are present without overt hemolysis, have also been reported. This underscores the necessity for routine post-transplant antibody screening [22].

### HSCT

HSCT is a crucial therapeutic approach for treating various malignant and non-malignant disorders. However, this strategy is often accompanied by a range of immune-mediated complications that can significantly impact patient outcomes. A unique characteristic of HSCT is its execution across the ABO blood group barrier, which involves the transfer of plasma, RBCs, and immunocompetent cells from the donor to the recipient. This process can lead to hemolytic anemia due to RBC incompatibility [75]. PLS is one such immune-mediated hemolytic complication that arises following allogeneic HSCT, primarily linked to minor or bidirectional ABO incompatibility. PLS is characterized by the production of antibodies, including anti-A/B or non-ABO antibodies, by the donor’s transplanted B cells, which subsequently target the recipient’s erythrocytes. Hemolytic events typically occur within 1–3 weeks post-transplantation and are frequently associated with GVHD and poor prognosis [9, 27, 35, 76].

Sudden-onset intravascular hemolysis presents with clinical features such as hemoglobinuria, jaundice, elevated LDH, and transfusion-refractory anemia [21, 77]. Symptoms generally appear 7–21 days following HSCT and correspond with donor lymphocyte engraftment. Diagnostic evaluation involves a positive DAT for IgG/C3d, the detection of donor-derived anti-recipient ABO or non-ABO antibodies in serum or eluates, and chimerism analysis to confirm the production of donor-derived antibodies [5, 78].

Risk factors for PLS include minor or bidirectional ABO incompatibility, which is more prevalent in unrelated or HLA-mismatched donors due to heightened immune responses [5]. The omission of post-transplant methotrexate (MTX) has been linked to an increased incidence of PTLD, while cyclosporine-based prophylaxis without MTX further exacerbates this risk. Moreover, peripheral blood stem cells may contribute to a higher susceptibility to PTLD, attributable to their elevated lymphocyte content compared to bone marrow [21, 76].

Concerning complications and prognosis, severe hemolysis may result in transfusion requirements that exceed the recipient’s RBC mass, as both native and transfused RBCs are subject to lysis. Additionally, post-liver transplantation (PLS) is associated with higher grades of acute GVHD and increased transplant-related mortality [79]. Laboratory findings typically reveal elevated LDH levels (> 1000 U/L), indirect hyperbilirubinemia (> 2 mg/dL), and a positive DAT due to donor-derived anti-A/B antibody-coated recipient RBCs. Retrospective studies involving 310 patients have underscored severe cases of transfusion-dependent anemia and fatal outcomes, highlighting the necessity for vigilant monitoring of high-risk populations.

The pathogenesis of PLS in patients receiving HSCT is primarily driven by donor B lymphocytes that produce anti-A/B antibodies, distinguishing it from autoimmune hemolytic anemia and plasma-derived hemagglutinin reactions. Current management strategies incorporate supportive care, including erythropoietin and antigen-matched transfusions, alongside immunosuppression, with rituximab achieving an 80% remission rate. However, challenges remain in standardizing HLA-matching documentation and clarifying transplant indications for hematologic diseases. Future research should focus on standardizing protocols for antibody profiling and HLA compatibility assessments to enhance risk stratification and therapeutic precision.

## Conclusion

PLS remains a significant challenge in transplantation medicine despite advancements in diagnostic and therapeutic strategies. Current management relies on risk stratification, including pre-transplant donor antibody screening (e.g., anti-ABO/Rh, anti-HLA) and cost-effective post-transplant monitoring (e.g., alternate-day DAT/LDH testing during days 5–30) to predict PLS onset, particularly in high-risk grafts such as liver and small bowel transplants. Although chimerism-based prediction holds theoretical promise, its clinical utility requires the further validation of cost-benefit ratios compared with conventional serologic monitoring. Novel therapeutic approaches, including complement inhibitors (e.g., ravulizumab) and B cell-targeted agents, demonstrate potentials for alleviating severe hemolysis and thrombocytopenia (e.g., efgartigimod NCT04188379). Nonetheless, the effectiveness of these treatments necessitates confirmation through large-scale clinical trials. Notably, live-donor transplants carry a marginally higher PLS risk, attributed to factors such as shared haplotype-driven immune priming and smaller graft mass altering lymphocyte equilibration dynamics. Three key priorities define PLS research:

1. Practical diagnostics: Refining accessible biomarkers (e.g., donor B-cell clonality and BAFF/proliferation-inducing ligand signalling profiles) to pre-emptively predict severe PLS.
2. Mechanism-driven therapies: Developing biologics that target donor lymphocyte subsets (e.g., BAFF-R inhibitors and anti-CD38 CAR-T cells) while preserving graft tolerance.
3. Global consensus frameworks: Establishing organ-specific guidelines through multinational registries to standardise monitoring and intervention protocols.

Translational challenges persist, particularly in balancing immunosuppression to prevent PLS while minimising the risks of infection and GVHD. In self-limiting cases, vigilant clinical observation remains the cornerstone as early over-treatment may be unwarranted. Collaborative efforts among transplant immunologists, hematologists, and computational biologists are pivotal for transforming PLS from a reactive complication to a preventable condition. Prioritising biomarker discovery, therapeutic innovation, and data-driven guidelines can improve graft survival and transplant recipients’ quality of life.
